# The hidden hedgehog of the pituitary: hedgehog signaling in development, adulthood and disease of the hypothalamic-pituitary axis

**DOI:** 10.3389/fendo.2023.1219018

**Published:** 2023-07-05

**Authors:** Yehan Bian, Heidi Hahn, Anja Uhmann

**Affiliations:** Institute of Human Genetics, Molecular Developmental Genetics, University Medical Center Göttingen, Göttingen, Germany

**Keywords:** hedgehog signaling, pituitary development, hypothalamic-pituitary axis, hormone production, pituitary disorders, pituitary tumors

## Abstract

Hedgehog signaling plays pivotal roles in embryonic development, adult homeostasis and tumorigenesis. However, its engagement in the pituitary gland has been long underestimated although Hedgehog signaling and pituitary embryogenic development are closely linked. Thus, deregulation of this signaling pathway during pituitary development results in malformation of the gland. Research of the last years further implicates a regulatory role of Hedgehog signaling in the function of the adult pituitary, because its activity is also interlinked with homeostasis, hormone production, and most likely also formation of neoplasms of the gland. The fact that this pathway can be efficiently targeted by validated therapeutic strategies makes it a promising candidate for treating pituitary diseases. We here summarize the current knowledge about the importance of Hedgehog signaling during pituitary development and review recent data that highlight the impact of Hedgehog signaling in the healthy and the diseased adult pituitary gland.

## 1 Introduction

More than 40 years ago, the two Nobel laureates Nüsslein-Volhard and Wieschaus reported about lethal *Drosophila melanogaster* (*D. melanogaster*) variants caused by gene mutations that altered segmental pattering of the larvae. One mutant was almost entirely covered with denticles. Due to its ‘hedgehog-like’ appearance, the responsible gene was named *hedgehog* (*hh*). Simultaneously, the *patch* locus was identified, which later turned out to contain the gene encoding for the Hh receptor patched (Ptch) (1). Subsequent studies in *D. melanogaster* identified further genes encoding components of the Hh signaling pathway including *suppressor of fused* (*sufu*) and *smoothened* (*smo*) (2, 3). Today, it is known that the HH signaling pathway is highly conserved between *D. melanogaster* and vertebrates and that it plays a fundamental role in embryonic patterning and development of most tissues and organs. This is recapitulated in the adult, where the pathway regulates tissue stem cells necessary for organ repair and maintenance. Thus, its dysregulation results in developmental defects and in a variety of cancers in vertebrates (reviewed in (4)).

The pituitary is one of the organs that depend on HH signaling (5–8). This so called ‘Master Gland’ represents one of the most important endocrine glands of the human body and acts as a mediator between the hypothalamus and peripheral organs by regulating the activity of most hormone glands. Thus, the pituitary controls essential vital body functions, e.g. growth, metabolism, reproduction, childbirth, lactation, stress responses and water/salt balance (9). Numerous studies revealed that HH signaling is essential to pituitary development by promoting patterning, cell proliferation and cell type determination in coordination with other signaling pathways (5–8, 10). Several recent studies indicate the involvement of HH signaling in pituitary hormone regulation as well as in the development of pituitary diseases (11–16). However, the role of HH signaling in the adult pituitary has remained largely unexplored. In this review, we summarize and discuss the impact of HH signaling in development and homoeostasis of the pituitary as well as in pituitary disorders (e.g. hypopituitarism, hyperplasia of the gland, neoplasms, hormone deficiencies or overproduction). Targeting HH signaling is an attractive and eligible therapeutic strategy, which is used for the treatment of a wide range of diseases (reviewed in (17)). It remains to be seen, whether this pathway also provides a target in the treatment of pituitary diseases.

## 2 The HH signaling pathway

### 2.1 Canonical HH signaling

**Figure 1:**
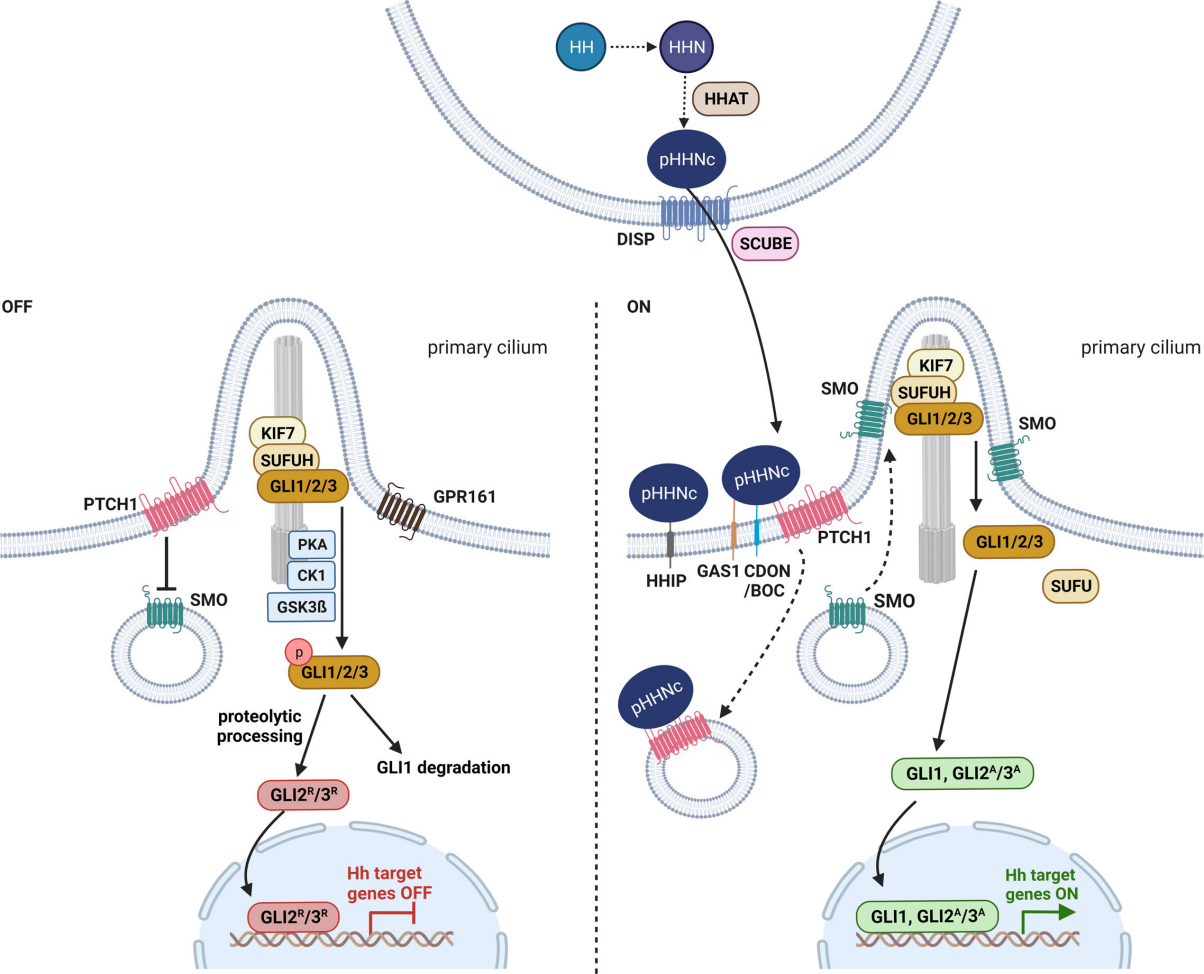
Hedgehog signaling in vertebrates. Before secretion, HH (hedgehog) is autoproteolytic cleaved into its N-terminal active form HHN and modified with cholesterol and HHAT (hedgehog acyltransferase)-mediated palmitoylation (pHHNc) (21, 22). pHHNc monomers are released from the membrane by DISP (dispatched) and SCUBE (signal peptide CUB-EGF-like domain-containing protein) (23). In the absence of pHHNc, PTCH1 (patched 1) accumulates in the primary cilium and inhibits SMO (smoothened) activity. GLI (transcription factor glioma associated oncogene) transcription factors are sequestered by KIF7 (kinesin-like protein 7) and SUFUH (suppressor of fused homolog) and phosphorylated by PKA (protein kinase A), GPR161 (G-protein coupled receptor 161), CK1 (casein kinase 1) and GSK3β (glycogen synthase kinase 3 β) kinases at the base of cilia, resulting in the degradation of GLI1 and the generation of GLI2/3 repressor (GLI2^R^/3^R^) forms. When pHHNc is present, it binds to its receptor PTCH1 and GAS1 (co-receptors growth arrest-specific protein 1), CDON (cell adhesion molecule-related/down-regulated by oncogenes) and BOC (brother of CDON), leading to the degradation of PTCH1 and the activation of SMO. Consequently, GLI dissociates from the SUFUH complex and acquires its active form (GLI1, GLI2^A^/3^A^) that promotes transcription of downstream target genes (19). Additionally, HHIP (hedgehog interacting protein) sequesters secreted pHHNc and negatively regulates HH signaling (24). The figure is based on (19, 20).

In vertebrates, three secreted HH ligands exist: sonic HH (SHH), indian HH and desert HH. Of these secreted factors, SHH is the most important and best-characterized one. Major components of the HH signaling pathway in vertebrates comprise the secreted HH ligands (e.g. SHH), the HH receptor and 12-transmembrane protein PTCH1, the seven-transmembrane G protein-coupled receptor and interaction partner of PTCH1 SMO, the suppressor of fused homolog (SUFUH) as a negative regulator of HH signaling as well as the glioma-associated oncogene (GLI) family of transcription factors (e.g. GLI1, GLI2, GLI3) (reviewed in (18–20)) (**Figure 1**).

Before secretion, the HH precursor protein is autoproteolytic cleaved to its N-terminal active form (HHN). Concomitantly, the HHN is modified with a cholesterol moiety at its C-terminus (HHNc) that allows tethering of HHNc to the outer cell membrane and further a palmitoyl group at its N-terminus (pHHNc) by HH acyltransferase (HHAT) (21, 22) (**Figure 1**). The latter lipidation is required for interaction with the HH receptor PTCH1 at the signal-receiving cell surface (22). Finally, the 12-transmembrane protein dispatched (DISP) and the cholesterol-binding protein SCUBE (CUB-EGF-like domain-containing protein) release pHHNc from the signaling cell (23) (**Figure 1**). Both occurrence and delivery of monomeric SHH via specialized filopodia (cytoneme) are regulated by a complex of SHH, DISP, CDON (cell adhesion molecule-related, down-regulated by oncogenes) and BOC (brother of CDON) (25) (reviewed in (26)). BOC, CDON and the membrane-bound protein GAS1 (growth arrest-specific gene 1) are also required for activation of HH signaling in the target cells (25, 27–29) (**Figure 1**). However, monomeric pHHNc can also self-associate to soluble multimers and get released from the membrane (30, 31). Furthermore, oligomeric pHHNc can interact with heparan sulphate chains of glypicans and form lipoprotein particles (32, 33). Alternatively, pHHNc can be released via exovesicles (34).

The released pHHNc molecules are then either captured by the HH interacting protein (HHIP), resulting in sequestration of the ligand, or by its receptor PTCH1 (24) that forms complexes with its coreceptors CDON, BOC or GAS1 on the surface of receiving cells (25, 27–29, 35, 36) (**Figure 1**). The binding of pHHNc to PTCH1 abolishes the inhibitory function of PTCH1 on HH signaling activity and leads to downstream pathway activation mediated by GLI transcription factors (24).

In vertebrates, both HH signal reception and intracellular HH signal transmission require the primary or sensory cilium, which is a specialized antenna-like organelle that functions in signal sensing and transduction. The primary cilium is a singular protrusion from the cellular surface that can be found on almost every vertebrate cell. It consists of nine microtubule doublets that extend from the membrane-docked mature centriole (basal body) and form the microtubule-based cytoskeletal core of the cilium, the so called axoneme. The axoneme is surrounded by a ciliary membrane, which is a specialized and distinct compartment of the plasma membrane. Molecules and proteins essential for ciliogenesis, maintenance and signaling of the cilium are transported within the cilium bi-directionally along the microtubules by intraflagellar transport. With respect to HH signaling, initial signal reception, pathway activation and transcription factor processing are linked to the primary cilium (reviewed in (37)).

In the absence of HH ligands, PTCH1 concentrates in the ciliary membrane and inhibits the entry of SMO into the cilia and thus blocks its activity (18, 38) (**Figure 1**). It is proposed that PTCH1 does not physically interact with SMO, but rather regulates SMO by restricting its access to cholesterol in the ciliary membrane (39). Upon inhibition of SMO, cytoplasmatic SUFUH and kinesin-like protein 7 (KIF7) restrain GLI transcription factor activity, and protein kinases (e.g. casein kinase 1, CK1; protein kinase A, PKA; glycogen synthase kinase 3 β, GSK3β; G-protein coupled receptor 161, GPR161) induce phosphorylation of the GLI proteins (**Figure 1**). Afterwards, GLI proteins are either completely degraded (GLI1) or marked by ubiquitination for partial cleavage into truncated GLI repressor forms (e.g. GLI2^R^, GLI3^R^) that translocate to the nucleus and repress the transcription of HH signaling target genes (**Figure 1**). This posttranslational proteolytic processing regulates the availability of GLI activator and repressor forms (reviewed in (19)). Since the proteolytic processing of GLI2 is not as efficient as that of GLI3, GLI2 is typically found in its full-length activator form and only occasionally occurs in a weak truncated repressor form (40). In contrast, GLI3 is efficiently processed and acts as a strong transcriptional repressor (40–42).

In the presence of HH ligands, pHHNc binds to PTCH1 and coreceptors CDON, BOC or GAS1. The pHHNc-PTCH1 complex is internalized and subjected to degradation by the lysosome (**Figure 1**). In turn, SMO molecules spread over the ciliary membrane and promote the dissociation of GLI from the cytoplasmatic SUFUH kinase complex by an unknown mechanism (19, 38). Subsequently, the GLI transcription factors are stabilized in their full-length activator forms (e.g. GLI1, GLI2^A^/3^A^) and translocate into the nucleus, where they promote HH signaling target gene expression (e.g. *HHIP*, *PTCH1*, *GLI1*) (reviewed in (19, 37)) (**Figure 1**). In turn, the main targets can regulate HH signaling activity in negative (e.g. HHIP and PTCH1) or positive feedback loops (e.g. GLI1, GLI2) (reviewed in (19, 43)).

### 2.2 Non-canonical HH signaling

Besides the canonical pathway, the HH signaling pathway is also activated non-canonically by mechanisms that are either SMO-independent or SMO-dependent but without GLI involvement. Shortly, the SMO-independent mechanisms are related to the potential of PTCH1 to interact with phosphorylated cyclin B1 and to recruit caspase-3 that leads to caspase-9 activation and apoptosis. Both interactions are independent of SMO and can be prevented by HH ligand binding. More complex are SMO-dependent, GLI-independent mechanisms. For example, SMO can use heterotrimeric G-proteins to promote cellular Ca^2+^ influx that affects the cytoskeleton, angiogenesis, cellular differentiation, proliferation, apoptosis and migration. For further details, please refer to some excellent reviews on this topic (reviewed in (19, 43–47)).

Finally, a plethora of other signaling pathways activates or inactivates the signaling pathway independently of the HH ligand. For example, active RAS/RAF/MEK/ERK (also known as MAPK pathway) as well as PI3K/AKT signaling are able to stabilize GLI proteins and to stimulate their activity via mitogen-activated protein kinase (e.g. MEK or MAP2K) (48–52). Furthermore, oncogenic RAS can – dependent on the cancer entity – also inhibit GLI via MEK and/or ERK (extracellular signal-regulated kinase) (53, 54). Additionally, WNT signaling is able to interfere with GLI signal transduction. β-catenin can stabilize GLI1 protein or induce *GLI1* transcription with the help of CRD-BP (coding region determinant-binding) (55) or sulfatase 2 (56), respectively. β-catenin can also physically interact with GLI1, which results in GLI1 degradation and inhibition of HH signaling (57). *Vice versa*, WNT signaling is also regulated by GLI proteins. Whereas active GLI2 can enhance β-catenin-dependent transcriptional activation (58), GLI3^R^ – in the absence of HH ligands – inhibits WNT signaling via interactions with β-catenin (59). Additionally, crosstalks between the HH/GLI and WNT/β-catenin signaling pathways on the level of SUFUH, CK1 and GSK3β have been described (60, 61). GLI transcription factors are also regulated by several other factors such as TP53 and NUMB, NOTCH signaling or microRNAs (reviewed in (43, 45)).

## 3 HH signaling in the pituitary gland

In human adults, the pituitary is a pea-sized organ and lies in the pocket-shaped *sella turcica* at the cranial base of the sphenoid bone. It is covered by a flat piece of *dura mater*, the *diaphragma sellae*. The pituitary borders laterally on the wall of the cavernous sinus and antero-inferiorly on the posterior wall of the sphenoid sinus. Antero-superiorly, it lies in close proximity to the optic chiasm (62, 63).

**Figure 2:**
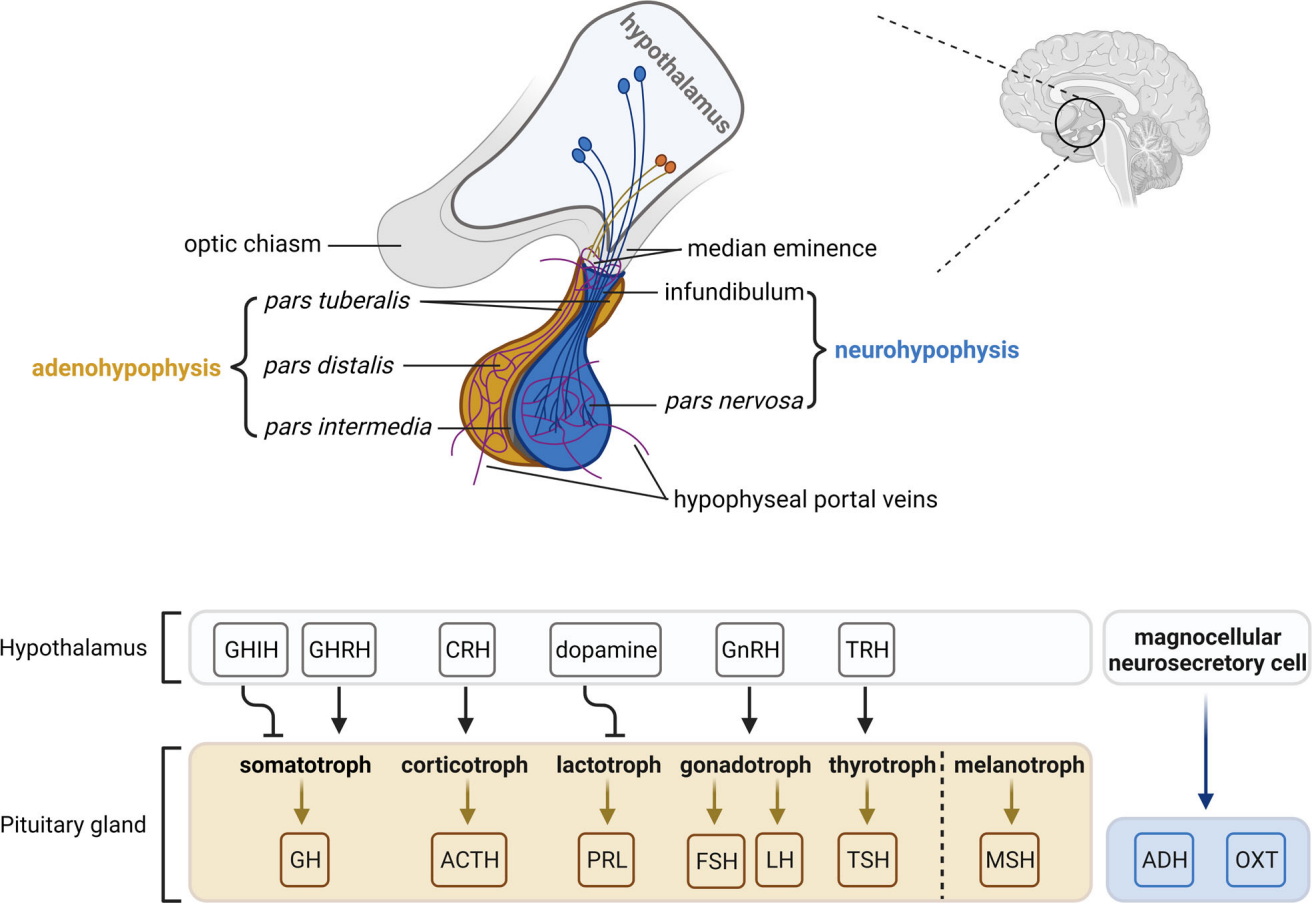
The hypothalamic-pituitary axis. The pituitary gland lies at the bottom of the hypothalamus and is composed of two functional components: the adenohypophysis consisting of the *pars tuberalis*, *pars distalis* and *pars intermedia*, and the neurohypophysis consisting of the infundibulum and *pars nervosa*. The *pars distalis* contains five endocrine cell types, which are somatotrophs, corticotrophs, lactotrophs, gonadotrophs and thyrotrophs that secrete growth hormone (GH), adrenocorticotropic hormone (ACTH), prolactin (PRL), follicle-stimulating hormone/luteinizing hormone (FSH/LH) and thyroid-stimulating hormone (TSH), respectively. The *pars intermedia* contains melanotrophs that secret melanocyte-stimulating hormone (MSH). Neurosecretory cells in the ventral nuclei of the hypothalamus secrete releasing/inhibitory hormones (growth hormone-inhibiting hormone, GHIH; growth hormone-releasing hormone, GHRH; corticotropin-releasing hormone, CRH; dopamine; gonadotropin-releasing hormone, GnRH; thyrotropin-releasing hormone, TRH) into hypophyseal veins at the median eminence and the infundibulum, which reach the adenohypophysis and regulate hormone activities. Magnocellular neurosecretory cells terminate in the *pars nervosa* and release oxytocin (OXT) and antidiuretic hormone (ADH) into the hypophyseal portal system. The figure is based on (9, 63).

Anatomically and functionally, the pituitary gland is composed of two distinct lobes: the adenohypophysis (anterior lobe) and the neurohypophysis (posterior lobe). The pituitary gland is connected with the superordinate hypothalamus via the infundibulum (infundibular stalk, pituitary stalk) and the hypophyseal portal system. The boundary between the adeno- and the neurohypophysis is called *pars intermedia* (intermediate lobe). In most animals, the *pars intermedia* is distinct from the fleshy, glandular adenohypophysis and the neural composition of the neurohypophysis. However, in humans, the intermediate lobe is only few cell layers thick and normally either very small or entirely absent in adulthood. As a result, it is often considered as a part of the adenohypophysis (reviewed in (9, 62, 63)) (**Figure 2**).

### 3.1 Embryonal pituitary development

The pituitary gland originates from two germinal layers. The adenohypophysis including the *pars intermedia* originates from an evagination of the oral ectoderm at the roof of the developing oral cavity (Rathke’s pouch; RP) and thus is of epithelial origin. The neurohypophysis develops from descendants of the neural ectoderm, precisely from the ventral hypothalamic floor of the ventral diencephalon (reviewed in (62, 64)). The development of the human pituitary starts approximately at the 4^th^ week of intrauterine development with the formation of the RP. During further growth of the pouch, the ventral diencephalon extends downwards to form the infundibulum. By the 5^th^ week of the gestation, RP comes into contact with the infundibulum. Around the 6^th^ to 8^th^ week, a constriction forms at the base of the RP and closes completely, thus separating it from the oral epithelium and standing in close contact with the infundibulum. Upon proliferation, cells of the anterior wall of the RP form the anterior lobe and later on, a small part of the anterior lobe subsequently grows up along the infundibulum, covers it and forms the *pars tuberalis*. Cells of the posterior wall of the RP develop to the *pars intermedia*. The lumen of the pouch obliterates and cells of the infundibulum form the pituitary stalk and the neurohypophysis. Finally, cell patterning and terminal differentiation occur within the adenohypophysis to form five principal and specialized endocrine cell types (62) (see details in the forthcoming chapter, **Figure 2**).

**Figure 3:**
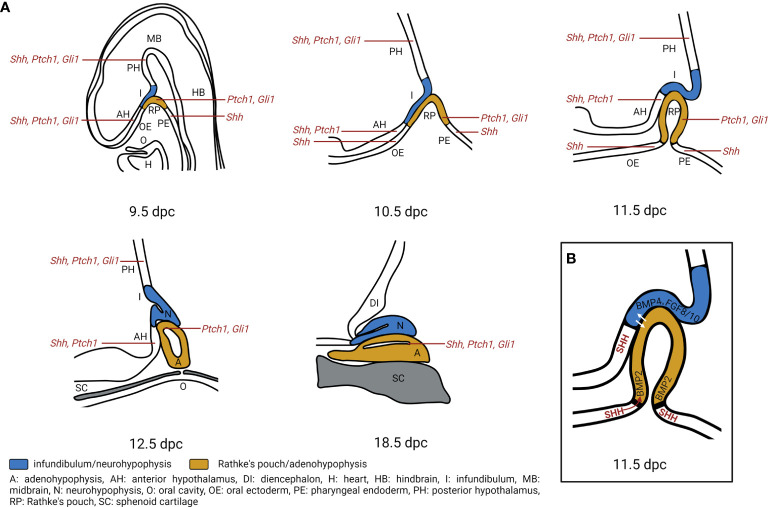
Hedgehog signaling regulates pituitary gland development. **(A)** Expression of *Shh*, *Ptch1*, *Gli1* transcripts during pituitary gland development. During murine embryogenesis *Shh* is expressed in the anterior hypothalamus (AH), posterior hypothalamus (PH), oral ectoderm (OE) and pharyngeal endoderm (PE) that is attached to the developing Rathke’s pouch (RP) from 10 dpc to 12.5 dpc (6, 7). *Ptch1* is expressed in the AH, PH, RP while *Gli1* is expressed in PH and RP from 10.5 dpc to 14.5 dpc. By 18.5 dpc, *Shh* is detected in sporadic single cells within the anterior pituitary, while *Ptch1*, *Gli1* are detected in the marginal zone of the pituitary (7). A, adenohypophysis; DI, diencephalon; H, heart; HB, hindbrain; I, infundibulum; MB, midbrain; N, neurohypophysis; O, oral cavity; SC, sphenoid cartilage. **(B)** Model of Hedgehog signaling regulation in pituitary gland development. SHH protein is expressed in the AH, together with BMP4. FGF8 and FGF10 that are expressed in the PH create an expression boundary within the hypothalamus that is essential for pituitary morphogenesis (10). Moreover, *Shh* expression marks a boundary between OE and RP which acts as an organizing center for ventral gene induction by inducing *Bmp2* expression in the ventral RP. The ventrodorsal SHH/BMP2 signaling and dorsoventral FGF8/FGF10 signaling putatively induce expression of temporally and spatially specific transcription factors and promote cell fate determination (5, 6). Additionally, hypothalamic SHH expression is required for cell specification and proliferation of LHX3^+^/LHX4^+^ progenitors in the RP (7).

As in humans, the adeno- and neurohypophysis in mice stem from the surface ectoderm and the neural ectoderm, respectively. The first sign of pituitary development occurs at 7.5 days post coitum (dpc) during murine embryogenesis. At this stage, a thickening of the ectoderm in the midline of the anterior neural ridge forms the hypophyseal placode that further develops into the RP between 9 to 10.5 dpc. By 10.5 dpc, when the definite RP has been formed, the neural ectoderm evaginates to form the infundibulum. The close and continuous contact of the neural ectoderm and the RP is essential for pituitary organogenesis. At 12.5 dpc, the pouch is completely separated from the underlying oral ectoderm. By 17 dpc, all parts of the murine pituitary have been developed. However, the lumen of the pouch persists as the pituitary cleft, separating the anterior and intermediate lobes in the mature gland (64) (**Figure 3A**).

#### 3.1.1 Signaling pathways and factors involved in pituitary development

Our current knowledge about embryonic development of the pituitary and the underlying mechanisms is mainly derived from mouse studies and phenotypes associated with human disorders that often share aspects with mouse models of defective pituitary development. Based on the respective observations, three general developmental steps can be distinguished in pituitary development: Firstly, initiation of pituitary organogenesis and formation of the RP, which are regulated by SIX homeodomain proteins (e.g. SIX1-6), paired-like homeobox proteins (e.g. HESX homeobox 1, HESX1; orthodenticle homeobox 2, OTX2; paired-like homeodomain transcription factor 1, PITX1; PITX2; PITX3), LIM homeodomain transcription factors (e.g. ISL LIM homeobox 1, ISL1; LIM homeobox 3, LHX3, LHX4) and by SOX2 (SRY-box transcription factor 2), β-catenin, NOTCH and SHH. Secondly, the proliferation and invagination of RP cells, which are controlled by bone morphogenic proteins (e.g. BMP2, BMP4), fibroblast growth factors (e.g. FGF8, FGF10, FGF18) and SHH. Thirdly, the lineage determination and cellular differentiation into endocrine cells, which is mediated by the stem cell marker PROP1 (homeobox protein prophet of PIT-1), PIT1 (or POU1F1, POU domain, class 1, transcription factor 1), GATA2 (GATA binding protein 2), NR5A1 (or SF1, steroidogenic factor 1) and TPIT (or TBX19, T-box transcription factor 19) (62, 64–66). Besides, additional factors expressed by the ventral diencephalon are implicated in regulation of pituitary formation. HESX1, SOX2, SOX3 and BMP4 expressed in the ventral diencephalon are regulators of RP progenitors, and OTX2, SIX6, SF1 (splicing factor 1), WNT5A, FGF8, FGF10 and FGF18 control further RP development (reviewed in (64, 67)). A summary of these transcription factors and signaling pathways as well as their roles in pituitary organogenesis are given in several excellent reviews (see (62, 64–67)). Here, we will focus on the role of HH signaling in the pituitary.

#### 3.1.2 Expression of HH signaling components in pituitary development

Early studies show an essential role of HH signaling in pituitary development. *Shh* is expressed in the ventral diencephalon and the lateral oral ectoderm at 10 dpc in mice, before the formation of the RP (5–8). However, as soon as the pouch appears, *Shh* expression is specifically excluded from the RP, resulting in a *Shh*-expressing and non-expressing boundary. The oral epithelium and the ventral diencephalon lose their *Shh* expression at 12 dpc or 14 dpc, respectively (6). Recent data furthermore show that *Shh* transcripts are expressed in the developing hypothalamus from 9.5 to 12.5 dpc and become restricted to an area of the ventricular zone of the 3^rd^ ventricle by 18.5 dpc (7) (**Figure 3A**). At 18.5 dpc *Shh* transcripts and Shh protein expression are also detectable in sporadic single cells within the adenohypophysis (7) (**Figure 3A**). *Gli1* and *Ptch1*, main target genes of HH signaling, are expressed throughout pituitary development. *Gli1* transcripts are expressed in the hypothalamus and in the RP between 9.5 and 12.5 dpc. *Gli1* expression is mainly detected in the dorsal region of the periluminal epithelium of the RP by 14.5 dpc and is restricted to the marginal zone of the adenohypophysis by 18.5 dpc (**Figure 3A**). *Ptch1* expression is detected in the hypothalamus except for the infundibulum from 9.5 to 14.5 dpc and is later restricted to the ventricular zone of the 3^rd^ ventricle by 18.5 dpc. In the RP, *Ptch1* is expressed between 9.5 dpc to 11.5 dpc. At 12.5 dpc, only a small region of the periluminal epithelium and by 18.5 dpc, only cells of the marginal zone express *Ptch1* (7) (**Figure 3A**). Similarly, in human fetuses, *SHH*/SHH transcript and protein have been identified in the anterior hypothalamus and the pharyngeal endoderm but not in the developing RP at Carnegie stage 15 (estimated postfertilization age of 36 days, 5^th^ week). *GLI1* expression is detectable throughout the developing hypothalamus and the RP (7). Altogether, the expression of HH signaling components in pituitary and adjacent regions indicates that the developing pituitary is competent to receive and respond to HH signaling. Most likely, SHH protein, that is produced in *SHH* transcript-positive regions adjacent to the developing RP (e.g. developing hypothalamus and pharyngeal endoderm), activates HH signaling in regions containing undifferentiated precursors/stem cells (e.g. RP, periluminal epithelium, marginal zone) (7).

### 3.2 The adult pituitary gland

#### 3.2.1 The hypothalamic-pituitary axis

The neurohypophysis is connected to the hypothalamus by the infundibulum. Magnocellular neurosecretory cells from the supraoptic and paraventricular nuclei of the hypothalamus project their axons to the neurohypophysis through the infundibulum (**Figure 2**). Magnocellular neurosecretory cells produce either oxytocin (OXT) and neurophysin I (NEU1) or vasopressin (antidiuretic hormone, ADH) and neurophysin II (NEU2) (**Figure 2**). OXT and ADH together with their respective carrier neurophysin proteins are stored and transported in neurosecretory granules of the axons called Herring bodies. Upon physiological stimuli like uterine stimulation during labor and suckling or increased osmolality of the plasma (e.g. hyponatremia), OXT-NEU1 or ADH-NEU2-containing vesicles are released from the axon terminals in the neurohypophysis into the systemic circulation via neurohypophyseal capillaries. In the bloodstream, OXT stimulates uterine contraction during labor and milk ejection from the breast, whereas ADH increases water reabsorption in the kidney and acts as a vasoconstrictor. Besides hypothalamic axons, the neurohypophysis also contains pituicytes that represent a subtype of non-neuronal glia cells assisting in storage and release of OXT and ADH (reviewed in (63, 68)).

Furthermore, the adenohypophysis is regulated by the hypothalamus via the hypophyseal portal system, which is a system of blood vessels. Neurosecretory axons in the hypothalamus terminate at the median eminence and release hypothalamic releasing and inhibitory hormones (e.g. corticotropin-releasing hormone, CRH; dopamine or prolactin release-inhibiting-hormone (PIH), growth hormone-releasing hormone, GHRH; gonadotropin-releasing hormone, GnRH; growth hormone-inhibiting hormone, GHIH; thyrotropin-releasing hormone, TRH). These releasing or inhibitory hormones reach the adenohypophysis via microcirculation and stimulate or inhibit the hormone secretion of their target endocrine cells, respectively (reviewed in (63, 68)) **Figure 2** *).*

As described in the afore-mentioned chapter, HH signaling is involved in the development of the hypothalamus-pituitary axis in that hypothalamic SHH expression promotes RP and pituitary formation (7, 10). However, little is known about the function of this pathway in the hypothalamus-pituitary axis in adulthood. Antonellis et al. reported expression of *Shh*, *Ptch1*, *Smo*, *Gli1*, and *Gpr161* in differentiated neurons of the adult mouse hypothalamus (69). In respond to fasting, mice show increased HH signaling activity in the ventromedial hypothalamus and the paraventricular nucleus of the hypothalamus involved in the regulation of feeding behavior and to a lesser extent in the cortex (69). Since the hypothalamic-pituitary axis participates in appetite, metabolism and feeding behavior (70), it is tempting to speculate that regulation of HH signaling in feeding behavior is carried out via this axis. Yet, further investigations are needed to illustrate the role of HH signaling in the adult hypothalamic-pituitary axis. Several sporadic and congenital diseases of the hypothalamic-pituitary axis are closely associated with a deregulated HH signaling pathway (see below, see **Tables 1**, **2**), making this research area particularly interesting.

**Table 1:** Human diseases associated with pituitary hypoplasia or deficiency.

congenital, syndromic hypopituitarism					
disease	cases per million	inheritance	hypothalamic-hypophyseal phenotype	causative mutations/variants	(potential) HH signaling …
**septo-optic dysplasia and its variants**	100	AD, AR, multigenetic	GHD, ACTHD, TSHD, GnRHD, DI	*ARNT2, BMP4, FGF8, FGFR1, HESX1, KAL1, OTX2, PAX6, PROKR2*, ***SOX2*** *, SOX3*, ***SHH***, ***(GLI2)*** (71–73)	*…* inactivation due to *SHH*, (*GLI2*) germline mutations (74)
**holoprosencephaly**	100	X-linked recessive, AD, AR	GHD, ACTHD, TSHD, DI	***CDON***, ***DISP1*** *, DLL1, FGF8, FOXH1*, ***GAS1***, ***GLI2*** *, NODAL*, ***PTCH1***, ***SHH*** *, SIX3, TGIF, TDGF1, ZIC2* (35, 75–84)	… inactivation due to *CDON, DISP1, GAS1, GLI2, PTCH1*, *SHH* germline mutations (35, 76–84)
***GLI2* mutations**	42 - 63	AD	GHD, ACTHD	***GLI2*** (85)	… inactivation due to *GLI2* germline mutations (85)
**hypopituitarism with cerebellar abnormalities**	2.7 - 6.4	AD	GHD panhypopituitarism	*LHX4* (75)	… is required for LHX3/LHX4 pituitary embryonic precursors (7)
**hypopituitarism with spine abnormalities**	1.5 - 5.4	AR	GHD, ACTHD, TSHD, FSHD/LHD panhypopituitarism	*LHX3* (75)	
**pituitary stalk interruption syndrome**	(~1,000 cases)	AD, AR	GHD, ACTHD, TSHD, FSHD/LHD	***CDON***, ***GPR161*** *, HESX1*, ***HHAT*** *, LHX4, OTX2, PROKR2*, ***PTCH1***, ***PTCH2*** *, SOX3* (86)	… inactivation due to *CDON, GPR161, HHAT, PTCH1, PTCH2* germline mutations (86)
**Pallister-Hall syndrome**	(~100 cases)	AD	GHD panhypopituitarism	***GLI3*** (87)	… inactivation due to *GLI3* germline mutations (87)
isolated hormone deficiencies					
**isolated GHD**	100 - 250	sporadic or 3-30% familial	GHD	*GH1, GHRHR*, ***GLI2***, *HESX1, LHX4, OTX2, SOX3* (75, 85)	… inactivation due to *GLI2* germline mutation (85)
**Kallmann syndrome**	20 - 100	X-linked recessive AD, AR, oligogen	LHD, FSHD	*CHD7, FGF8, FGFR1, KAL1, PROK2, PROKR2, SOX10*, ***WDR11*** (75)	… inactivation due to *WDR11* germline mutations (88, 89)
**CHARGE syndrome**	66 - 86	AD	GHD, ACTHD, TSHD, FSHD/LHD	***CHD7*** (90)	… inactivation due to *CHD7* mutations (91)
**Wolfram Syndrome**	est. 1.3 - 2	AR	DI	***WFS1*** (92) (rarely *CISD2*)	… inactivation due to *Wfs1* mutation (93)

ACTHD, ACTH deficiency; AD, autosomal dominant; ADHD, ADH deficiency; AR, autosomal recessive; DI, diabetes insipidus; est., estimated; FSHD/LHD, FSH deficiency/LH deficiency; GHD, GH deficiency; GnRHD, GnRH deficiency; MSHD, MSH deficiency; OXTD, OXT deficiency; PRLD, PRL deficiency; TSHD, TSH deficiency.

Wiedemann-Steiner syndrome or PHACES (Posterior fossa anomalies, Hemangioma, Arterial anomalies, Cardiac anomalies, and Eye anomalies Syndrome), which are also associated with pituitary hypoplasia or deficiency, are not listed. Genes associated with HH signaling are highlighted in bold.

**Table 2:** HH signaling and other sporadic tumors of the sellar region.

hypothalamic tumors						
disease	subtype	cases per million	origin	phenotype/ tumor hormone production	frequently mutated genes (94)	potential HH signaling …
**hypothalamic endocrine tumors**	**neurocytoma**	(26 cases)	neuronal cells	(ADH, GHRH)	*PHF14* (95)	… inhibition results in reduced proliferation of neurocytoma (96) … might be associated with malignant progression of neurocytoma (97)
	**pilocytic astrocytoma**	4.8	astrocytes	central precocious puberty (GH)	*BRAF, CRAF, FGFR1, NF1, KRAS, PTPN11* (98)	… activation in pilocytic astrocytoma (99)
**PPT**	**pituicytoma**	very rare	pituicytes	no tumoral endocrine activity (possible hormone production of surrounding tissue)	*BRAF, CBL, FGFR1*, ***GLI2*** *, HRAS, MAP2K2, NF1*, ***PTCH1*** *, PTEN, TERT* promoter and other (100–102)	… deregulation due to *PTCH1* mutation/variant (101)
	**spindle cell onkocytoma**	very rare		no tumoral endocrine activity; high incidence of hormonal deficit		… activation due to nuclear GLI2 (case report) (102)
	**sellar epen-dymoma**	(12 cases)	heterotopic ependymal cells	(DI, PRL/panhypopituitarism possible)	unknown	… activation due to GLI1, GLI2 overexpression in ependymoma (103)
other neoplasms of the sellar region						
**cranio-pharyngeoma**	**adaman-tinous**	0.5 - 2	remnants of RP cells	no tumoral endocrine activity, CPHD	*CTNNB1* (104)	… overactivation in adamantinous craniopharyngioma (104)
**germinoma**	–	1.2 - 7.2*	primordial germ, pluripotent embryonic or neural stem cells	DI, central precocious puberty	*AKT1, CBL*, ***GLI3*** *, KIT, KRAS, NRAS*, ***PTCH1***, ***SHH*** (105–108)	… activation due to gain of *SHH* and *GLI3* and loss of *PTCH1* (1/6 cases) (105)
**suprasellar meningioma**	–	(1% of sellar masses)	*diaphragma sellae* or *tuberculum sellae*	(PRL possible)	*AKT1, KLF4, NF2, PIK3CA*, ***SMO*** *, TRAF7* (109, 110)	… activation due to activating *SMO* mutations (109, 110)
**chordoma**	–	0.1-0.8	intraosseous notochordal remnants	no tumoral endocrine activity; coexisting PitNETs	LOH 1p36.13 and 7q33 (111) *PBRM1* (112)	… activation due to *SHH*, *PTCH1*, *SMO*, *SUFU*, *GLI1* expression (113, 114) … overactivation (high Gli1 expression) correlates with poorer prognosis (115)
**chondroma/chondrosacroma**	–	(0.15% of all intracranial tumors)	embryo/adult cartilage cells	CPHD possible	*CDKN2A, COL2A1*, ***GLI1*** *, IDH1, IDH2*, ***HHIP***, ***PTCH1***, ***RUNX2***, ***SUFU*** (116, 117)	… activation in chondroma (118, 119)

*in Western countries, higher in Asia; CPHD, combined pituitary hormone deficiency; DI, diabetes insipidus; PPT, posterior pituitary. tumor.

Not listed are gangliocytoma, granular cell tumor, papillary craniopharyngioma, pituitary blastoma and Rathke’s cleft cysts. Genes associated with HH signaling are highlighted in bold.

#### 3.2.2 Structure and function of the adenohypophysis

The adenohypophysis is the major organ of the endocrine system that hormonally regulates physiological processes like stress response, growth, reproduction, and lactation. It is composed of three regions: the *pars distalis*, the *pars tuberalis* (*infundibularis*) and the *pars intermedia* (reviewed in (63)) (**Figure 2**).

The *pars distalis* comprises the major part of the adenohypophysis and represents the region where most of the pituitary hormone production occurs. There are five types of endocrine cells: somatotrophs, corticotrophs, lactotrophs, gonadotrophs and thyrotrophs that secrete growth hormone (GH), adrenocorticotropic hormone (ACTH), prolactin (PRL), luteinizing hormone (LH) or follicle-stimulating hormone (FSH), and thyroid-stimulating hormone (TSH), respectively (**Figure 2**). The distribution of these endocrine cells in humans follows a certain pattern with somatotrophs and lactotrophs clustering in the lateral region, corticotrophs in the central regions, thyrotrophs in the anterior regions and gonadotroph scattered throughout the *pars distalis* (reviewed in (62)). Together with the non-endocrine folliculostellate (FS) cells, these endocrine cells form a 3D network that allows homotypic and heterotypic interactions between the cells, at least in rodents and fishes (120–125) (reviewed in (126)). Additionally, the *pars distalis* and the marginal zone – a single layered epithelium between the *pars distalis* and the *pars intermedia* – contain SOX2^+^ non-endocrine cells that exhibit stem cell properties since they self-renew and can differentiate into all five endocrine cell types. They contribute to organ homeostasis and probably are also in tumorigenesis due to their potential to differentiate into all mature hormone-producing lineages and their auto- and paracrine signaling (reviewed in (127, 128)). The adenohypophyseal hormone production and release are tightly controlled by hormones of the hypothalamus and from other endocrine organs in negative and positive feedback loops (e.g. liver, adrenal gland, thyroid gland etc.). For example, the release of GH is controlled by the hypothalamic hormones GHRH and GHIH, but also by the liver-born insulin-like growth factor-1 (IGF-1), GHIH of delta cells of the digestive system and by thyroid hormones (tri-iodothyronine (T3) and thyroxine (T4)) (reviewed in (62, 63)) (**Figure 2**).

The *pars tuberalis* represents a part of the sheath extending up from the *pars distalis.* It consists of gonadotrophs, thyrotrophs and FS cells that form an incomplete collar around the infundibulum/pituitary stalk of the neurohypophysis. Research of the last decade strongly indicates that the *pars tuberalis* is a major player in the regulation of seasonal reproduction and photoperiodicity, since it lies in direct proximity to the suprachiasmatic nuclei and expresses melatonin receptors and specific clock genes (reviewed in (62, 63, 129)).

The *pars intermedia*, as described above, represents the thin cell layers that mark the boundary between the adeno- and the neurohypophysis (**Figure 2**). In humans, it consists of colloid-filled cysts as well as of melanotrophs, corticotrophs, gonadotrophs and thyrotrophs (reviewed in (62)).

Somatotrophs constitute about 30-40% of all adenohypophyseal cells. They produce and release GH that exerts its effects in almost all tissues. GH promotes linear growth by stimulating protein synthesis and transport of amino acid through the cells. In the liver, GH induces the production of the somatomedins IGF-1 (somatomedin C), IGF-2 (somatomedin A) and vitronectin (VTN, with a somatomedin B-domain), which stimulate cell division and cellular proliferation. For example, somatomedins enhance T-cell proliferation, increase the absorption of calcium from the intestine and reduce calcium loss through urination, stimulate hepatic glycogenolysis to raise levels of glucose in the blood, increase the growth of soft and skeletal tissues and increase the uptake of non-esterified fatty acids by the muscle. The release of GH from somatotrophs in the adenohypophysis is positively controlled by hypothalamic GHRH. Negative feedback loops that inhibit GH secretion are mediated by high circulating GH concentrations and by liver-born IGF-1 that stimulates hypothalamic GHIH secretion (reviewed in (63, 130)).

Corticotropic cells and melanotrophs express pro-opiomelanocortin (POMC) which is the precursor protein for ACTH and β-lipotropin (β-LPH) in corticotrophs and for melanocyte-stimulating hormones (α-MSH, β-MSH, γ-MSH) in melanotrophs. In response to hypothalamic CRH stimulation, corticotrophs secrete ACTH that stimulates the adrenal cortex to produce cortisol. Via a negative feedback, cortisol inhibits the release of hypothalamic CRH and thus hypophyseal ACTH secretion. A functional role of β-LPH has not been proved so far. However, β-LPH is cleaved into β-endorphin and γ-LPH that is the precursor of β-MSH. In fact, corticotrophs produce and secrete β-endorphin acting as endogenous opioid neuropeptide that reduces stress and maintains homeostasis. Conversely, melanotrophs of the *pars intermedia* produce and secrete α-MSH, β-MSH and γ-MSH. These proteins are derived from different regions of POMC and bind with different preferences to different melanocortin receptors. Circulating α-MSH induces the release of melanin pigment in skin melanocytes during human fetal life and upon sun exposure in adulthood. In lower vertebrates, such as fish or amphibians, Msh from the *pars intermedia* induces skin darkening in response to changes in background color, whereas Msh released from hypothalamic neurons controls feeding and energy expenditure (reviewed in (63, 130)).

Lactotrophs comprise about 20% of all cells in the adenohypophysis. They produce and release PRL, which induces growth and development of the breast and maintains lactation in women. The production and secretion of PRL is primarily controlled by the hypothalamus that releases dopamine, which predominantly inhibits PRL release. Upon nipple stimulation of lactating women, sensory neurons are activated causing an inhibition of the dopamine release and thus an unopposed release of PRL. During pregnancy, the pituitary gland may increase by approximately 30% due to lactotroph hyperplasia (reviewed in (63, 130)).

Gonadotrophs release either FSH or LH. In females, FSH stimulates growth and development of follicles in the ovary and secretion of estrogens by the mature follicle, while LH triggers ovulation and stimulates the secretion of progesterone by the corpus luteum. In males, LH stimulates Leydig cells in the testes to secrete testosterone, whereas FSH stimulates Sertoli cells to secrete androgen-binding proteins that initiate spermatogenesis. In both males and females, these gonadotropic hormones help to stimulate the maturation of primordial germ cells. LH, FSH as well as human chorionic gonadotropin (hCG) and TSH (see below) are glycoprotein hormones. All these hormones are heterodimers consisting of non-covalently associated alpha (glycoprotein hormones, alpha polypeptide encoded by the *CGA* gene) and beta subunits (β-FSH, β-LH, β-hCG, β-TSH). The production and release of FSH and LH from the adenohypophysis are controlled by GnRH produced by the hypothalamus. Additionally, steroid sex hormones (e.g. estrogen) inhibit FSH and LH secretion via negative feedback loops (reviewed in (63, 130)).

Thyrotrophs compose around 5% of the adenohypophysis. They produce and release the glycoprotein hormone TSH that is controlled by the hypothalamic TRH. TSH functions as a stimulator of the thyroid gland to produce and release the hormones T3 and T4. In a negative feedback, T4 inhibits TSH secretion (reviewed in (63, 130)).

FS cells are non-endocrine cells of the adenohypophysis. They have a stellate shaped, follicular morphology with long cytoplasmic processes (123). Their lysosome content and their large number of microvilli on the apical side suggest that FS cells are phagocytic (131). Additionally, the FS cell mesh forms a 3D network, in which FS cells and endocrine cells reside and communicate via gap junction-mediated calcium wave propagation (123). Moreover, FS cells control adenohypophyseal cell function via cytokines and growth factors and modulate inflammatory response feedbacks by secreting inflammatory cytokines (reviewed in (132, 133)). Due to the lack of specific marker protein expression, the identification of FS cells remains challenging. So far, studies show that S100b and GFAP (glial fibrillary acidic protein) are strongly expressed in early, newly formed FS cells (134). Besides, other papers report that cytokeratin, vimentin, fibronectin (135) or SOX2 (16) are expressed in FS cells. However, whereas GFAP (136) and vimentin expression (137) suggest a neuroectodermal origin of FS cells, keratin or SOX2 expression rather implies epithelial-like (138) or stem cells-like characteristics. In addition, FS cells show high annexin A1 (ANXA1) levels, which is comparable to non-endocrine cells of the hypothalamus (139). Since glucocorticoids increase ANXA1 expression in FS cells, it is hypothesized that ANXA1^+^ FS cells subsequently regulate corticotrophs, which express ANXA1-associated G protein coupled receptors, via a paracrine mechanism (139–141). In the *pars tuberalis*, FS cells are localized in close association with GnRH-containing axon terminals, which regulate seasonal reproduction and photoperiodicity. Thus, FS cells may also be involved in transmission of photoperiodic stimuli to the reproductive system (63). Interestingly, the stem cell marker SOX2 is co-expressed with S100 protein in FS cells of adult mouse pituitaries (142) and in GH-producing pituitary neuroendocrine tumors (PitNETs) (143). Taking together, a bulk of data support the idea that FS cells function as sustentacular (mechanical/chemical) supporters for pituitary endocrine cells (144, 145). However, because these cells also show stem cell-like characteristics, the exact role of FS cells still remains mysterious.

#### 3.2.3 HH Signaling in the healthy adult adenohypophysis

Vila et al. were the first scientists, who evaluated the potential role of HH signaling in the adult human adenohypophysis (13, 14). Their studies revealed that SHH and GLI1 are exclusively expressed in corticotrophs. Besides, they reported that PTCH1 is expressed in gonadotrophs and thyrotrophs, while PTCH2 (a PTCH1 paralog) is expressed in corticotrophs, somatotrophs and some lactotrophs (13, 14). Our recent data confirmed the SHH expression in corticotrophs of the human adenohypophysis. However, we additionally found SHH expression in somatotrophs and lactotrophs (11). In the adenohypophyseal tumor cell lines AtT-20 (corticotroph) and GH3 (lactotroph/somatotroph), expression of all four HH signaling components (SHH, PTCH1, PTCH2 and *Gli1*) is detected simultaneously (13, 14). This contrasts the data from human adenohypophysis samples that show no PTCH1 expression in corticotrophs and no GLI1 and PTCH2 expression in lactotrophs/somatotrophs (11, 13, 14). In addition, lineage-tracing experiments showed that approximately 2/3 of all somatotrophs and 2/3 of all FS cells descend from GLI1-expressing cells in the murine adenohypophysis (12), indicating a role of HH signaling in somatotrophs and FS cells. Together, these data are somewhat inconclusive. However, recent single cell RNA sequencing data revealed the expression of *GLI1*, *GLI3* and *GAS1* in human SOX2^+^ pituitary cells (146) and of *Ptch1*, *Gli1/2/3* in FS cells and of *Shh* in gonadotrophs in chicken pituitary glands (147). Additionally, we re-analysed data from Cheung et al. (148) who sequenced single cells of adult male mouse pituitary glands and identified stem cells and hormone-producing cell types. Using this data set, we found that a subpopulation of murine somatotrophs expresses *Gli1* while corticotrophs, gonadotrophs and lactotrophs cells do not. Together, these data support the conclusion that SOX2^+^ stem cells and/or FS cells as well as somatotrophs (at least in mice) express markers of active HH signaling. Therefore, HH signaling may be involved in pituitary homeostasis and/or tissue regeneration.

Furthermore, functional studies revealed that activation of HH signaling with recombinant SHH protein in AtT-20, GH3 and cultured rat pituitary cells results in increased ACTH, GH and/or PRL secretion, respectively (13, 14). A simultaneous treatment with SHH and CRH even increased ACTH production of AtT-20 and rat pituitary cells, whereas GLI1 inhibition reduces the CRH-induced stimulation of *Pomc* transcription (13). These data support the idea that active HH signaling directly regulates ACTH, PRL and GH secretion, and CRH signal transduction in the adult pituitary. Compared to these studies, our data rather hint towards an indirect hormone regulation by HH signaling in the adult adenohypophysis. We found that HH signaling activation in murine pituitary explants induces *Acth*, *Gh*, *Prl* and *Cga* expression (11), whereas a corticotroph-specific *Ptch1* or *Smo* depletion *in vivo* does not (12). Moreover, neither corticotrophs nor lactotrophs show active HH signaling in adult mice (12). Interestingly, the FS cell line TtT/GF is responsive to HH signaling activation. Treatment with the SMO-aginist SAG of the FS cell line TtT/GF results in SMO localization to primary cilia and an enhanced expression of *Gli1* and *Gli2* (12). Remarkably, the supernatant from SAG-stimulated TtT/GF cells induces GH production and release in GH3 cells, which are mediated by the neuropeptide vasoactive intestinal protein (VIP) (12). The facts that GLI1 expression overlaps with the stem cell and FS cell marker SOX2 (11, 16) and that HH signaling activation induces the proliferation of SOX2^+^ cells in the *pars distalis* (11) further substantiate the assumption that HH signaling regulates pituitary hormone production indirectly via FS cells. This hypothesis is also supported by conditional *Ptch1* depletion and concomitant activation of HH signaling in FS and CD45^+^ hematopoietic cells, which has a functional impact on multiple pituitary endocrine cells, including gonadotrophs and thyrotrophs (15). Nevertheless, future studies are needed to examine whether this concept is transferable to the human adenohypophysis and potentially also to corticotrophs and lactotrophs.

## 4 HH signaling in the diseased pituitary

Given that major components of HH signaling pathway are expressed during hypothalamic-pituitary development and in the adult pituitary, it is not surprising that the deregulation of this pathway is closely related to developmental defects or diseases of the hypothalamus and/or pituitary gland.

### 4.1 Hypothalamus and pituitary developmental defects caused by deregulated HH signaling

#### 4.1.1 Lessons from animal models

The earliest hint for HH signaling involvement in development of pituitary diseases came from studies of *Shh* null mouse embryos that die just before or at birth and show forebrain malformations. These developmental defects result in a phenotype resembling holoprosencephaly in humans and in an agenesis of the pituitary gland (149). Similarly, *Gas1* (27, 150) and *Cdon* null embryos (27, 151, 152) display microforms of holoprosencephaly and agenesis of the pituitary. Craniofacial developmental defects are also detected in *Boc* null mutants (153). These results support an essential role of HH signaling in pituitary development (see chapter ‘*Expression of HH Signaling Components in Pituitary Development*’ and **Figures 3A, B**). Hypothalamus-specific SHH depletion during murine embryogenesis results in pituitary hypoplasia and absence of the optic disc, which are key features of a rare developmental disorder in humans called Septo-optic dysplasia (SOD; please see below for detailed description) (10). These embryos develop a highly dysmorphic infundibulum that fails to protrude correctly from the diencephalon and is shifted anteriorly within the brain. Additionally, the formation of the anterior pituitary in these embryos is affected. The RP fails to pinch off the oral ectoderm and also shifts anteriorly into the brain (10). Likewise, a decrease in the SHH regulators SOX2 and SOX3 reduces SHH levels, expands FGF10 and BMP4 expression in the ventral diencephalon and thus may be the cause of SOD development (10). When SHH is depleted in a more restricted area of the anterior hypothalamus, RP development is completely arrested and an infundibulum fails to form. This results in complete pituitary agenesis, severe craniofacial defects, and telencephalic and hypothalamic abnormalities (7).

Moreover, HH signaling most likely promotes cell proliferation and cell-type determination in pituitary development. The before-mentioned hypothalamus-specific SHH depletion in mice results in a significant reduction of somatotrophs, corticotrophs and thyrotrophs (10). When SHH is depleted in the mouse anterior hypothalamus, *Lhx3/Lhx4* expression is lost in the RP epithelium, which is accompanied by a progressive cell fate transformation of the epithelium. Besides, most of the hypothalamic factors controlling pituitary function (e.g. GHRH, GHIH, OXT, POMC) are lost or severely reduced in these mice, suggesting a loss of neuronal differentiation due to interrupted HH signaling (7). Similarly, a HHIP-mediated inhibition of HH signaling during very early stages in the oral ectoderm (prior to and during RP formation) results in a cystic rudiment of the pituitary gland that lacks the expression of ventral cell type markers (*Bmp2*, *Gata2*, *Pou3f4*) and of the terminal differentiation markers POMC and β-TSH (6). The study also implicates that *Shh* expression marks a boundary between the *Shh*-expressing oral ectoderm and the non-expressing RP. This boundary functions as an organizing center for ventral gene induction by inducing *Bmp2* expression in the ventral RP and thus directing patterning and proliferation of the gland. Treier et al. furthermore hypothesized that ventrodorsal SHH/BMP signaling and dorsoventral FGF8/FGF10 activity lead to the induction of temporally and spatially restricted transcription factors expression and promote cell-fate determination (6) (**Figure 3B**). However, HH signaling inhibition (pharmacologically via the SMO-inhibitor cyclopamine or in *smu*/*smu* mutant embryos) during different zebrafish developmental stages revealed that HH signaling may also play a role even before invagination of the oral ectoderm and during early patterning of the gland (154). In contrast, HH signaling inactivation via a *Gli2* mutation results in a variable loss of the adeno- and neurohypophysis in mice (155, 156). The *Gli2* loss does not affect the overall patterning of the pituitary even not when *Gli2* is specifically depleted before RP closure in cells of the anterior neural ridge and the early telencephalon (156). Its loss rather impacts on diencephalic expression of BMP4 and FGF8 and on proliferation of pituitary progenitor cells in the RP, which finally results in defective development of the neurohypophysis and of hormone-producing cells of the murine adenohypophysis (156). Interestingly, and in contrast to GLI2, neither GLI1 nor GLI3 depleted pituitaries show any abnormality. However, excessive production of GLI3, as in *Shh* null and *Hhip*-overexpressing embryos, may be responsible for severe pituitary phenotypes such as pituitary hypoplasia (6, 7, 10, 156). Together, these results show that inactivation of HH signaling results in a hypoplastic or missing pituitary.

*Vice versa*, an activation of the pathway during hypothalamic-pituitary formation results in a hyperplastic gland and/or hyperpituitarism in mice. Thus, ectopic SHH overexpression under control of the CGA-promoter in RP cells leads to an increased organ volume and elevated cell numbers of thyrotrophs and of LH-producing gonadotrophs (6). *Ptch1* depletion in *Hesx1* cell lineage, which marks the anterior neural plate including the prospective telencephalon, anterior hypothalamus and RP epithelium, results in HH signaling activation in RP cells from 10.5 dpc. This leads to increased proliferation of RP progenitors and development of a hyperplastic adenohypophysis due to an enlarged SOX2^+^ stem cell compartment (7). However, cell commitment (at 14.5 dpc) and differentiation of hormone-producing cells (at 18.5 dpc) in these embryos are unchanged (7). Similarly, overactive HH signaling in the ventral diencephalon due to a *Tbx3* (T-box transcription factor 3) depletion results in reduced *Fgf10* and increased *Ptch1* expression in the infundibulum at 10.5 dpc. This goes along with hyperproliferation of the ventral diencephalon, hypoplasia of the adenohypophysis and lack of evagination of the infundibulum and thus of the neurohypophysis (157). Indeed, FGF10^+^ progenitors give rise to the neurohypophysis, whereas HH signaling orchestrates the development of the infundibulum and the RP and promotes the growth and differentiation of progenitors of the adenohypophysis, at least in chicks (158). Finally, pharmacological stimulation of HH signaling of a three-dimensional, ES-cell derived adenohypophysis tissue results in self-formation of RP-like structures *in vitro*. Moreover, these RP-like aggregates develop into functional corticotrophs or somatotrophs and lactotrophs upon manipulation of other signaling pathways (159).

Altogether, animal studies suggest that HH signaling plays a crucial role in pituitary development via regulation of patterning, cell proliferation and cell-fate determination. Moreover, HH signaling in both RP cells (6) and the developing hypothalamus (7, 157, 158) seems to be important for pituitary formation.

#### 4.1.2 Human diseases

Similar to the above-mentioned animal models, mutations in the HH signaling pathway in humans result in defects of the pituitary. In fact, inactivating mutations in HH signaling components have been identified in subsets of patients suffering from SOD, Pallister-Hall syndrome (PHS), holoprosencephaly (HPE), isolated or combined pituitary hormone deficiency (CPHD), congenital hypopituitarism without HPE, or pituitary stalk interruption syndrome (PSIS) (reviewed in (75, 76, 160)) (**Table 1**).

SOD is a rare developmental disorder and is characterized by optic nerve hypoplasia combined with midline abnormalities of the brain and pituitary hypoplasia/hypopituitarism (71). Thus, SOD patients can suffer from isolated GH deficiency (GHD), CPHD and/or diabetes insipidus. Whereas the most common disease-causing mutations are found in *HESX1*, *SOX2* and other genes, recently a causative variant in *SHH* in a SOD family has been identified (74). Mice with a conditional *Shh* deletion in the hypothalamus recapitulate this SOD phenotype (see above) (10). Moreover, the impact of HH signaling in SOD is substantiated by the fact that SOX2 induces SHH expression (10, 91) and directly regulates *HESX1*/*Hesx1* transcription (161).

Similarly, the rare PHS is related to mutational inactivation of HH signaling during embryonal development. This autosomal dominant inherited disease is caused by frameshift/nonsense or splicing mutations in the second third of the *GLI3* gene (from nucleotides 1998-3481) that lead to an exclusive expression of the GLI3^R^ (162, 163). This results in developmental defects like mesoaxial polydactyly, hypothalamic hamartoma, and sometimes in GHD and panhypopituitarism (reviewed in (164)).

Furthermore, variants in *PTCH1* (77), *CDON* (35), *GLI2* (78), *DISP1* (79), and *GAS1* (80) have been detected in patients with HPE. Interestingly, approximately 17% of familial HPE are associated with inactivating *SHH* mutations or *SHH* haploinsufficiency (76, 81–84). The phenotypes of HPE range from most severe forms that include cyclopia and loss of the pituitary gland to less severe forms with milder symptoms such as hypotelorism, a single midline incisor, and hypopituitarism (165–167). Thus, HPE patients often suffer from several endocrine problems. These include GHD or ACTH deficiency (165, 168), which result in short stature or adrenal hypoplasia, respectively. In addition, loss-of-function mutations in *GLI2* are also within the HPE spectrum, but are associated with a distinctive phenotype. The primary features of this phenotype are defective anterior pituitary formation and pan-hypopituitarism (78). This is similar to the developmental defects found in *Gli2* mutant mice (155, 156) (see above). However, more recent reports rather associate *GLI2* variants with polydactyly, pituitary deficiency and subtle facial phenotypes (169–173). In addition, *GLI2* mutations are apparently also responsible for congenital hypopituitarism without HPE or for CPHD (i.e. impaired GH production combined with an impairment of at least one other anterior pituitary hormone) (85, 170, 174, 175). The mutational spectrum of these phenotypes includes *GLI2* variants that result in loss of GLI2 function, or at least has a negative impact on the proteins function (174, 175). CPHD patients with GLI2 loss can suffer from deficiencies in somatotrophs, thyrotrophs and gonadotrophs, maldescended neurohypophysis or ADH deficiency (170, 174, 176). However, *GLI2* mutations have also been detected in some patients with isolated GHD (see below) (75, 85).

Finally, mutations in *PTCH1, PTCH2* (177), *GPR161*, *HHAT* and *CDON* have been found in PSIS patients (86, 178, 179). PSIS is characterized by a combination of three specific findings on magnetic resonance imaging, which are an interrupted pituitary stalk, an absent or ectopic posterior pituitary and anterior pituitary hypoplasia. Besides these characteristics, PSIS is also associated with other midline and ophthalmic abnormalities and variable endocrine disorders such as hypoglycemia, growth failure, or CPHD (86, 178, 179).

It also should be mentioned that hypopituitarism is a symptom of other syndromes that are caused by variants in genes that may regulate HH signaling activity. These syndromes include Prader-Willi (75), Williams (180), Kabuki (181), Axenfeld-Rieger (182, 183), Johanson-Blizzard (184) or Oliver-McFarlane syndromes (185). However, the description of the mechanisms by which the respective genes may regulate HH signaling activity in pituitary development is unknown or out of the scope of this review. In addition, several isolated hormone deficiencies are linked to deregulated HH signaling. For example, some patients with isolated GHD show *GLI2* mutations (75, 85). Similarly, the development of central hypogonadism in Kallmann or CHARGE syndromes has been linked to HH signaling. Thus, some Kallmann syndrome patients show mutations in *WDR11* (WD repeat domain 11) that modulate HH signaling activity by regulating GLI3 processing (88, 89). Likewise, 65% of CHARGE syndrome patients show variants of CHD7 (chromodomain helicase DNA binding protein 7) that physically interacts with SOX2 to regulate a set of common target genes including *GLI3* (91). Additionally, some forms of central diabetes insipidus may be linked to deregulated HH signaling. For example, *WFS1* (Wolframin ER transmembrane glycoprotein) mutations, which are found in 95% of Wolfram syndrome patients, lead to GLI1 degradation in *in vitro* analyses (93) (**Table 1**).

### 4.2 HH signaling in sellar neoplasm

It is well known that mutational activation of HH signaling results in a broad range of tumor entities (44, 177–179). Currently three basic mechanisms for mutational activation of the pathway in cancers are proposed. The first mechanism is mutation-driven and ligand-independent and was detected in patients suffering from the inherited Nevoid basal-cell carcinoma syndrome (NBCCS) (86, 180). This disease is caused by heterozygous *PTCH1* mutations resulting in aberrant HH signaling activity in the absence of the HH ligand (86, 180). Besides developmental defects, NBCCS patients show an increased risk for a variety of tumors (e.g. medulloblastoma, basal cell carcinoma). Thus, the detection of *PTCH1* mutations in this syndrome was the first link between HH signaling and cancer. Besides *PTCH1* mutations, the mutation-driven subtype of HH signaling activation also includes activating mutations in *SMO*, *GLI1* or *GLI2* or inactivating mutations in *GAS1*, *CDON* or *GLI3*. The second type of activation is ligand-dependent and autocrine. This mechanism is characterized by an overexpression and oversecretion of HH ligands by the respective tumor, which results in an autocrine stimulation of tumor cell growth. Similarly, the third mechanism is characterized by an overexpression and oversecretion of HH ligands by the tumor cells. However, in this case the ligand-dependent activation is paracrine, in that the tumor-born HH ligands activate HH signaling in tumor and/or in stromal cells (reviewed in (181)). HH-associated malignancies are usually characterized by high expression levels of downstream targets like GLI1 and/or GLI2 as indicators for a pathologically overactive pathway. However, whereas the role of HH signaling in classical HH-associated tumors such as medulloblastoma and basal cell carcinoma is well known, that of neoplasms of the pituitary or the sellar region is far from clear.

#### 4.2.1 Neoplasm of the sellar region

Neoplastic lesions in the sellar region can be classified as PitNETs (182), hypothalamic (endocrine) tumors, tumors of the neurohypophysis (posterior pituitary tumors, PPTs) and other tumors. The development of tumors in the sellar region is either sporadic, phenotypic for congenital disorders like hypothalamic hamartoma or associated with syndromes that predispose to neuroendocrine tumors (NETs). These syndromes encompass neurofibromatosis type 1 (NF1), Lynch syndrome, multiple endocrine neoplasia 1 (MEN type 1 and 2), Von Hippel-Lindau (VHL) syndrome, McCune-Albright syndrome, DICER1 syndrome, Carney complex (CNC), Succinate dehydrogenase deficiency, familial isolated pituitary adenoma (FIPA) and familial infantile gigantism (X-LAG syndrome) (reviewed in (147, 184))

PitNETs comprise approximately 15% of all primary sporadic brain tumors and are the most common pituitary disease (186). Most PitNETs are slow-growing and benign. A metastatic behavior of PitNETs is exceptionally rare (< 0.5% of PitNETs) (187). PitNETs are frequently hormone-secreting, which indicates an origin from hormone-secreting cells of the adenohypophysis. They are classified based on their histological expression pattern of certain transcription factors, such as TPIT (or TBX19), PIT1 (or POU1F1) or SF1 (NR5A1). TPIT^+^ PitNETs account for approximately 15% of PitNETs. Because these tumors secrete ACTH (corticotroph), which results in excess of ACTH, the phenotype of the patients resembles that of Cushing disease. PIT1^+^ PitNETs are subdivided into tumors, which release GH and/or CGA (somatotroph), PRL (lactotroph), TSH (thyrotroph) or GH together with PRL and/or TSH (mammasomatotroph, mature plurihormonal, immature PIT1-lineage). Over 33% of resected PitNETs are SF1^+^ and secrete FSH and LH (gonadotroph) (reviewed in (188–190)). PitNETs that express TPIT, PIT1 or SF1 on protein level and one or more hormones at clinically irrelevant levels, are further classified as silent PitNETs (191). Together with null cell adenoma (see below), they were historically categorized into the group of non-functioning pituitary adenomas (NFPAs). However, according to the newest WHO classification guideline, null cell adenoma together with unclassified plurihormonal tumors now account for the fourth class of PitNETs, the undefined PitNETs. Undefined PitNETs are negative for TPIT, PIT1 and SF1, but whereas unclassified plurihormonal tumors secrete ACTH, GH, PRL, TSH, FSH and/or LH, null cell adenomas do not (reviewed in (188–190, 192)). However, silent and null cell PitNETs can also lead to hormonal deficiencies due to compression of the surrounding pituitary areas (**Table 3**).

**Table 3:** HH signaling and PitNETs.

pituitary neuroendocrine tumors (PitNETs)/adenomas						
disease	subtype	cases per million	origin	phenotype/ tumor hormone production	frequently mutated genes (94)	potential HH signaling …
**TPIT^+^ PitNETs**	**corticotroph**	~150	supposedly hormone-secreting cells	ACTH	*AIP, ATRX, BRAF1*, ***DICER1***, ***FGFR2***, ***GNAS*** *, MLH1*, ***NR3C1*** *, PABPC1, SH2, TP53*, ***TSP-1***, ***USP8***, ***USP48*** (94, 100, 193)	… activation in ACTH PitNETs (11) … dependent pathologies are non-additively influenced by Dicer1 (194) … activation by FGFR2 signaling (195–197) … activation due to *GNAS* null mutations (198–200) … inhibition by NR3C1 signaling (201) … activation by *TSP-1* null mutation (202) … activation by USP48-mediated GLI1 stabilization (203, 204) … activation by USP8 (205)
**PIT1^+^ PitNETs**	**somatotroph**	>150		GH and/or αGSU	*AIP*, ***GNAS*** *, GRB101*, ***PRKAR1A***, ***MEN1*** *, MAX, SDHD* (94, 100, 193, 206–208)	… activation in GH PitNETs (11) … activation due to *GNAS* null mutations (198–200) … inactivation by PKA signaling (209) … activation due to *MEN1* mutation (210)
	**lactotroph**	n. a.		PRL	*AIP, HMGA2*, ***PRKAR1A***, ***MEN1*** *, KIF5A, MAX, SDH, SF3B1* (94, 206–208)	… activation in PRL PitNETs (11) … inactivation by PKA signaling (209) … activation due to *MEN1* mutation (210)
	**thyrotroph**			TSH	*ASTN2, CWH43, R3HDM2, SMOX, SYTL3*, ***THRb*** *, ZSCAN23* (211)	… regulation byTHR (212)
**NFPA**	–	n. a.		none	*CDKN2A, ENC1, FAM90A1, ING2*, ***MEG3*,** *MLH3, MSH5, MSH6, PI3K* (213)	… activation by MEG3 inhibition (214)

NFPA, non-functioning pituitary adenoma; PitNET, pituitary neuroendocrine tumor.

Not listed are mammasomatotroph, mature plurihormonal, immature PIT-1 lineage, acidophil stem cell, gonadotroph and undefined PitNETs. Genes associated with HH signaling are highlighted in bold.

Sporadic PitNETs frequently show epigenetic changes (215–217), whereas common mutations are quite rare (reviewed in (218)). Nevertheless, subsets of sporadic corticotroph, somatotroph and lactotroph PitNETs can harbor activating mutations in *GNAS* (*guanine nucleotide binding protein, alpha stimulating*), which result in constitutive activation of cyclic AMP signaling (reviewed in (193)). The tumors can also show *AIP* (*aryl hydrocarbon receptor interacting protein*) mutations, whose role in tumorigenesis is still unclear (reviewed in (219)). Furthermore, some GH- or PRL-producing PitNETs carry mutations in *MEN1, CDKN1B* (*cyclin-dependent kinase inhibitor 1B*), *MAX* (*MYC associated factor X*) or *PRKAR1A* (*protein kinase CAMP-dependent type I regulatory subunit alpha*) and ACTH-secreting tumors can harbor *USP8* (*ubiquitin specific peptidase 8*), *USP48*, *DICER1, MLH1* (*MutL homolog 1*) or *MSH2* (*MutS homolog 2*) mutations. In addition, pituitary stalk hemangioblastomas can harbor *VHL* (*Von Hippel-Lindau tumor suppressor*) mutations (reviewed in (193)) (**Table 3**).

A small subset of PitNETs is also caused by germline mutations associated with familial endocrine syndromes, whose disease-causing loci are identical to those found in sporadic PitNETs (reviewed in (218)) (**Table 4**). For example, the autosomal dominant inherited MEN types 1, 4 and 5 are caused by heterozygous inactivating mutations in *MEN1*, *CDKN1B* and *MAX*, respectively (reviewed in (193)). Depending on the mutated gene, MEN1, MEN4 and MEN5 patients develop PitNETs in 30%, 42% and 80% of cases, respectively, which are mostly PRL- or GH-releasing tumors. Postzygotic activating mutations in *GNAS* cause McCune-Albright syndrome that leads to somatotroph/corticotroph PitNETs and pituitary hyperplasia in 6% and 14% of patients, respectively (235). *GNAS* mutation are also found in GH-secreting PitNETs of patients suffering from the autosomal dominant inherited NF1 syndrome (231). 2% of all PitNETs are caused by the autosomal dominant FIPA syndrome that predisposes to functioning/non-functioning PitNETs (236). 20% of FIPA patients have heterozygote inactivating *AIP* mutations, whereas the genetic cause in the remaining patients is unknown (193). Patients suffering from the autosomal dominant inherited Carney complex CNC develop pituitary hyperplasia, which results in the formation of lactotroph or somatotroph PitNETs in 20% of the cases (237, 238). The molecular cause is heterozygous inactivating mutations of *PRKAR1A* or *PRKAR1B* genes (232) (reviewed in (193)). Moreover, congenital genetic variants of the *DICER1* gene cause the extremely rare DICER1 syndrome that predisposes to the formation of pituitary blastoma, ACTH-dependent hypercortisolism and corticotroph PitNETs (233, 234). Furthermore, germline mutations in *VHL* in the Von-Hippel-Lindau syndrome result in various benign and malignant tumors of the central nervous system including pituitary stalk hemangioblastomas (227). Finally, *MLH1* and *MSH2* mutations are responsible for Lynch syndrome, which leads to the formation of aggressive ACTH-secreting PitNETs (222, 223).

**Table 4:** Human syndromes predisposing for PitNETs.

syndromes predisposing to PitNETs						
disease		cases per million	inheritance	phenotype (tumor hormone production)	frequently mutated genes	(potential) HH signaling …
**Neurofibromatosis syndrome type 1**		300 -400	autosomal dominant	optic pathway gliomas, high GH levels, rarely PitNETs, empty sella, hypopituitarism	*NF1* (reviewed in (220))	… is involved in tumorigenesis of NF1 tumors (221)
**Lynch syndrome**		330	autosomal dominant	PitNETs (ACTH)	*EPCAM, MLH1, MLH3, MSH2, MSH6, PMS2*, ***TGFBR2*** (222, 223)	… inhibition by TGFBR2 in cancer (224)
**MEN**	**type 1**	33 - 100	autosomal dominant	***MEN1*** (193)	*MEN1* (193)	… activation due to activating *Men1*/*MEN1* mutation (210, 225)
	**type 2**	28	autosomal dominant	***RET*** (193)	*RET* (193)	… activation due to activating *RET* mutations (226)
**von Hippel–Lindau syndrome**		27	autosomal dominant	pituitary stalk hemangioblastomas	***VHL*** (227)	… activation due to *VHL* mutation (228)
**hypothalamic hamartoma**		est. 1 - 20	sporadic/5% Pallister Hall syndrome	clusters of small neurons intermixed with glia and large neurons, GHD, TSHD, posterior pituitary dysfunction	***GLI3*, *OFD1, SOX2*** (161, 229, 230)	… inactivation due to *SOX2, GLI3* mutations (161, 230) … mutations enriched in hamartomas (161, 230)
**McCune-Albright**		1 - 10	postzygotic (non-germline)	14% pituitary hyperplasia, 6% PitNETs (GH, PRL),	***GNAS*** (postzygotic, activating) (231)	… activation due to *GNAS* null mutations (198–200)
**Carney complex**		(160 cases)	autosomal dominant	pituitary hyperplasia, 20% PitNETs (GH/PRL)	***PRKAR1A***, ***PRKACB*** ***(*** 232)	… inhibition by PKA signaling (209)
**DICER1 syndrome**		(1,000 cases)	potential autosomal recessive	pituitary blastoma PitNETs (ACTH)	***DICER1*** (233, 234)	… dependent pathologies are influenced by Dicer1 (194)

est., estimated; MEN, multiple endocrine neoplasia.

Not listed are MEN syndrome type 4 and 5, MEN-like syndrome, Familial isolated pituitary adenoma, Familial infantile gigantism and Succinate dehydrogenase deficiency. Genes associated with HH signaling are highlighted in bold.

Hypothalamic tumors are subdivided into gangliocytoma, neurocytoma and pilocytic astrocytoma (low-grade optic/hypothalamic glioma), whereas PPTs are categorized into pituicytoma, granular cell tumors (GCT), spindle cell oncocytoma (SCO), sellar ependymoma (SE). Other tumors of the sellar region are craniopharyngiomas, germinomas, suprasellar meningiomas, chordoma, chondroma, Rathke’s cleft cysts (RCC) and hamartoma. Except for PitNETs, tumors of the sellar region have different cellular origins such as the craniopharyngeal duct (craniopharyngiomas), or arachnoid cells (meningiomas) or are primordial germ cell remnants (germinomas). Nevertheless, pituicytomas, GCT, SCO and SE, that together account for only 0.5% of all sellar tumors, probably represent a spectrum of a unique neuropathological entity that most likely originate from pituicytes of the neurohypophysis (183). Whereas hypothalamic endocrine tumors can lead to acromegaly, Cushing disease or precocious puberty due to excess GHRH, CRH or GnRH/PRL production, respectively (reviewed in (239)), PPTs as well as all other tumors of the sellar region are hormonally inactive. With exception of the malignant chordoma and chondroma, hypothalamic tumor as well as PTTs are benign neoplasms (**Table 2**).

#### 4.2.2 HH signaling in PitNETs

Several of the genetic variants in sporadic and syndrome-associated PitNETs are linked to HH signaling (**Tables 3**, **4**). GLI1 is known to inhibit the mismatch repair (MMR) pathway (reviewed in (240)) whose members MLH1 and MSH2 are mutated in sporadic corticotroph PitNETs (193) and also are the reason for Lynch syndrome that is associated with aggressive ACTH-secreting PitNETs (222, 223). Moreover, USP8 (205, 241) and USP48, which are mutated in corticotroph PitNETs (193), are positive regulators of HH signaling activity (203, 204). USP8 prevents SMO ubiquitylation and increases HH signaling activity by promoting SHH-induced cell surface accumulation of SMO (205, 242), whereas USP48 interacts with GLI1 in the nucleus and thereby protects GLI1 from proteasome-dependent degradation. The resulting GLI1 protein stabilization enhances the proliferation and colony formation ability of glioma cells *in vitro*. Additionally, active HH signaling induces USP48 expression via a positive feedback loop by GLI1 binding to the *USP48* promoter (203).

In contrast, *GNAS*, *MEN1*, *PRKAR1A* and *DICER1*, which are mutated in sporadic PitNETs (**Table 3**) and in familiar diseases predisposing for PitNETs (**Table 4**), are known HH signaling repressors (198–200, 209, 210, 225). Thus, both GNAS and DICER1 act as tumor suppressors in HH-dependent medulloblastoma formation in mice (194, 200). Similarly, VHL, which is mutated in pituitary stalk hemangioblastoma of VHL patients, decreases GLI1-mediated promoter transactivation and thus inhibits expression of HH signaling target genes (228).

Together, these data suggest a functional role of HH signaling in PitNETs. However, the observation that USP8 and USP48 rather positively regulate HH signaling, whereas GNAS, MEN1, PRKAR1A, DICER1 and VHL rather negatively regulate this signaling pathway in PitNETs, does not allow a definitive conclusion, whether it is active or inactive HH signaling that is involved in formation of these tumors. In fact, the first report about the HH signaling activation status in PitNETs showed that ACTH-secreting Cushing tumors produced low SHH protein, whereas its receptors PTCH1 and PTCH2 were detectable at variable levels (14). Subsequent *in vitro* analyses revealed that SHH stimulation of corticotroph, somatotroph or lactotroph PitNETs inhibits cell proliferation and elevates secretion of ACTH, GH or PRL, respectively. Thus, it was hypothesized that active HH signaling is necessary for hormone production, whereas loss of HH signaling activity results in tumor formation in the adult pituitary (14). However, if this hypothesis turns out to be true, PitNETs would be one of the rare tumor entities that depend on an inactive HH signaling cascade for their development or at least for their maintenance, as reported for colorectal cancer (243). In addition, this hypothesis contradicts two observations: firstly, it interferes with the growth-activating role of HH signaling during pituitary development (78, 156, 170, 244, 245); secondly, this assumption would imply that PitNETs need active HH signaling for hormone production. However, this is obviously not the case since Cushing tumors are generally SHH negative (14). In fact, a growth-activating role of HH signaling in PitNETs is underlined by a study by Lampichler et al., which demonstrates that GLI1, as a major indicator for active HH signaling, is present in 87% of all PitNETs and significantly correlates with the expression of the stem cell marker SOX2 and the proliferation marker MKI67 (246). Moreover, purmorphamine-induced HH signaling activation increases cell viability of AtT-20 cells, whereas inhibition of GLI1 results in cell death (246). Since *GLI1*/GLI1 expression was found in almost all human pituitary tumors studied, whereas *SHH* mRNA was present in few tumors samples (246) (see also (14)), the authors hypothesized that GLI1 expression in pituitary tumors is stimulated in a ligand-independent way and plays an important role in tumorigenesis, while the reduction in SHH expression is likely a compensatory mechanism.

Contrarily, we found that almost all PitNETs express *SHH*/SHH, which strongly and significantly correlates with *GLI1* mRNA expression levels. Remarkably, this is identical with classical HH-associated malignancies, which include the highly aggressive medulloblastoma and the benign basal cell carcinoma (see above). Since ACTH-, GH- and PRL-immunopositive PitNETs show the highest SHH and *GLI1* levels, we propose that HH signaling activity may play a role in formation and/or maintenance of these pituitary tumor subtypes (11) as it is known for classical HH-associated tumors. Similarily, Yavropoulou et al. found higher PTCH1, PTCH2, GLI1 and GLI3 levels in somatotroph PitNETs compared to normal pituitary samples. However, they did not observe any hint for HH signaling activity in PRL-secreting PitNETs and NFPA, which even showed lower PTCH1, PTCH2, GLI1, GLI3 and HHIP levels than control tissue (247). All these studies do not answer whether the expression level of HH-related genes is pathogenetically associated with the development of the repective PitNET or simply reflects a compensation mechanism for tumor growth initiated by other factors (247). Nevertheless, several recent publications support the assumption that active HH signaling plays a central role in PitNETs. For example, functional analysis of differentially expressed genes between PitNETs and normal pituitaries identified the HH signaling pathway as one of the three pathways significantly enriched in the tumors (248). In addition, PitNETs with a proliferation MKI76-index above 3% express significantly higher *GLI1*/GLI1 levels than tumors with a proliferation index below 3% or normal pituitaries (249). In line with this observation, activation of HH signaling also induces the proliferation of *in vitro* cultured PitNETs cells, whereas inhibition of the pathway inhibits it (249). Moreover, recent single cell transcriptomes of 21 PitNETs compared to 3 normal pituitary glands show that the SOX2^+^ stem-like cell compartment of PitNETs (tumor stem cells) is enriched for the expression of genes involved in HH signaling and expresses higher *GLI1* and *GLI2* levels (250).

Together, these functional analyses of recent years strongly reinforce a tumor-supportive role of HH signaling in PitNETs. However, additional studies are needed to substantiate this assumption and to test therapeutic possibilities by targeting the HH signaling pathway in this tumor entity.

#### 4.2.3 HH signaling in hypothalamic endocrine tumors and PPTs

In terms of the genetic landscape of sporadic hypothalamic tumors and PPTs, the majority of the tumors show activating mutations in the RAS/RAF/MEK/ERK signaling pathway. For example, gangliocytoma show mutations in *BRAF*, *KRAS*, *RAF1*, *NF1* (*neurofibromin 1*) and *CDKN2A* (*cyclin dependent kinase inhibitor 2A*) (251) and tumors of the glia in *FGFR*, *HRAS*, *BRAF* and *NF1* (100). Interestingly, hypothalamic tumors or PPTs also harbor mutations in HH signaling components. Recently, a *PTCH1* variant has been described in pituicytoma (101). Loss of *PTCH1* together with copy gains of *SHH*, *SMO* and *GLI3* have been reported in 1/6 germinomas (105). Additionally, some chordoma show activating mutations in HH signaling components (115). These studies indicate that HH signaling may also play a role in tumorigenesis in some of these tumor entities. Indeed, histological examination of hypothalamic tumors or PPTs also reveal HH signaling activity in several of these neoplasms. GAB1 (GRB2 associated binding protein 1) that can serve as a histological read-out for active HH signaling (252), is upregulated in malignant neurocytoma (97). Moreover, *PTCH1* and *GLI1* expression are elevated in 45% of analyzed pilocytic astrocytoma (99) and a strong nuclear GLI2 signal, indicative for active HH signaling, has been detected in a case of SCO (102). Similarly, active HH signaling may be present in SE, since ependymoma in general overexpress GLI1 and GLI2 (103). Active HH signaling has also been detected in chordoma and adamantinomatous craniopharyngioma (ACP) (104, 113, 115, 118, 119, 253–256). Surprisingly, HH signaling apparently has anti-tumorigenic properties in the latter tumor entity. Thus, inhibition of HH signaling increases the proliferation of human APC cells and decreases the median survival of APC-bearing mice due to premature development of highly proliferative and vascularized undifferentiated tumors (257). Similarly, hypothalamic hamartomas, that are clusters of small neurons intermixed with glia and large neurons, often are associated with PHS caused by exclusive GLI3^R^ expression. They also contain *SOX2* mutations (161, 229, 258, 259) that lead to inefficient HH signaling (91). This indicates that HH signaling inactivation may not necessarily contribute to the formation this tumor entity (**Table 2**).

Together, these data suggest that HH signaling in APC and hypothalamic hamartomas has a rather tumor-suppressive function, whereas in hypothalamic endocrine tumors and PPTs it may have an oncogenic function. However, HH signaling activity in gangliocytoma, pilocytic astrocytoma (low-grade optic/hypothalamic glioma), GCT, suprasellar meningioma or RCC has not been reported so far. It will be highly interesting to see whether HH signaling also plays a functional role in these tumor entities.

## 5 Conclusions and future directions

Research of the last twenty years has greatly improved our knowledge about the impact of HH signaling in the embryonic and adult pituitary. Animal experiments show that cells in the developing pituitary are competent to receive and respond to HH signaling, and that HH signaling promotes patterning, cell proliferation and terminal differentiation during pituitary organogenesis. In line with these observations, aberrant HH signaling activity is frequently found in human developmental disorders associated with pituitary malformations or hormone deficiencies, indicating that the HH signaling cascade plays an important role during pituitary development in humans as well. Very recent data further suggest that HH signaling is also active in adulthood and regulates pituitary homeostasis and hormone production. In addition, HH signaling seems to have a tumor-supportive role for PitNETs and PPTs. However, additional studies are needed to support these findings and to test whether HH signaling may be an intervention target for treatment of pituitary diseases.

So far, a large number of HH signaling pathway inhibitors have been developed and have entered clinical trials as anti-cancer agents. To date, two SMO inhibitors (i.e. vismodegib, sonidegib) have been approved by the US Food and Drug Administration (FDA) and by the European Medicines Agency (EMA) for treating locally advanced basal cell carcinoma. Both drugs are currently tested in various clinical trials for other tumor entities either as a single agent or in combination with other anticancer drugs (reviewed in (17)). Thus, vismodegib or sonidegib are currently tested in combination with either the focal adhesion kinase inhibitor GSK2256098, the AKT-inhibitor capivasertib or the CDK4*/*6*-*inhibitor abemaciclib in the treatment of meningioma (NCT02523014). They are also used in combination with gemcitabine, the CDK4*/*6*-*inhibitor ribociclib, the BRAF-inhibitor trametinib or the cytokine filgrastim for treatment of brain tumors (like astrocytoma, meningioma, ependymoma) in children and young adults (NCT03434262). However, to date treatments of pituitary neoplasms with HH signaling inhibitors have been only reported in mouse models. For example, Ding et al. showed that inhibition of HH signaling by vismodegib blocks tumor growth of xenotransplanted sterol carrier protein 2-overexpressing GH3 cells (249). Currently, dependent on their tumor size and hormone production, PitNETs are subjected to surgery and/or radiation therapy. Medications can help to block excess hormone secretion. Thus, corticotroph PitNETs that cause Cushing syndrome are treated with drugs controlling cortisol production in the adrenal gland such as ketoconazole, mitotane and metyrapone. Somatotroph or lactotroph PitNETs are treated with somatostatin analogs or with the dopamine agonists cabergoline and bromocriptine, respectively (reviewed in (260)). Although most of pituitary tumors are benign, some of them show an aggressive behavior and are either locally invasive, rapidly growing or even metastasizing (189, 261). In addition, some PitNETs recidivate (262–264). For these tumors, the therapy is rather experimental and definitely needs improvement (260). Thus, there is hope that a better understanding of the role of HH signaling in the pituitary will contribute to better treatment options in case of disease.

## Author contributions

All authors listed have made a substantial, direct, and intellectual contribution to the work and approved it for publication.
